# Real-Life Benefit of Omalizumab in Improving Control of Bronchial Asthma During COVID-19 Pandemic

**DOI:** 10.7759/cureus.17268

**Published:** 2021-08-17

**Authors:** Polliana Mihaela Leru, Vlad Florin Anton

**Affiliations:** 1 Family Medicine, Carol Davila University of Medicine and Pharmacy, Bucharest, ROU; 2 Internal Medicine, Colentina Clinical Hospital/Carol Davila University of Medicine and Pharmacy, Bucharest, ROU; 3 Internal Medicine, Colentina Clinical Hospital, Bucharest, ROU

**Keywords:** asthma control, biologic therapy, covid-19 pandemic, omalizumab, real-life benefit

## Abstract

Biologic therapy is recommended by Global Initiative for Asthma (GINA) guidelines in asthma patients not controlled with maximal inhaled therapy corresponding to GINA step 4. Omalizumab is an anti- immunoglobulin E (IgE) monoclonal antibody and the first biological available for the add-on treatment of severe allergic asthma, approved by Food and Drug Administration (FDA) in 2003. Diagnosing and managing asthma patients during coronavirus disease 2019 (COVID-19) pandemic since early 2020 has been challenging, mainly due to the risk of contracting COVID-19 disease and to the limited access to hospital care and pulmonary function tests. We report a case of a 52-year-old female patient, diagnosed with adult-onset asthma in 2018, who was first referred to the Allergy Department of our hospital in January 2019 for dyspnea, wheezing, and worsening cough. Despite continuous inhaled therapy and good inhalation technique, she had frequent asthma symptoms, requiring short courses of oral corticosteroids (CS). Physical examination and pulmonary function tests on admission revealed broncho-obstructive syndrome and laboratory tests showed mild inflammation and high total serum IgE. She continued to have two moderate-severe exacerbations after stepping up to maximal inhaled therapy plus oral montelukast and theophylline, according to GINA step 4. By the end of 2019, we additionally started omalizumab, which resulted in prompt clinical benefits and resolution of asthma symptoms. Given the ongoing COVID-19 pandemic limiting in-person visits, virtual follow-ups indicated adequate control of his symptoms, as proved by asthma control test and no need for hospital presentation.

## Introduction

Asthma is one of the most common chronic obstructive pulmonary diseases, characterized by bronchial hyperresponsiveness and variable airflow limitation, due to chronic airway inflammation. Asthma is still a significant cause of morbidity and mortality, representing a challenging pathology in clinical practice, with an estimated prevalence between 300 and 400 million people globally [1]. 

Although the majority of asthmatic patients can achieve disease control with standard inhaled therapy, up to 10% of patients have severe asthma that remains poorly controlled, despite good adherence to treatment with high doses of inhaled corticosteroids (ICS) and long-acting bronchodilators [2]. The economic burden of the medical care for severe asthma is four times higher compared to controlled asthma patients in Europe [3].

Asthma is not a uniform disease, but rather a heterogeneous one, with multiple phenotypes that are caused by a variety of pathophysiological mechanisms or endotypes. Intensive research and international collaboration have led to better characterization of distinct inflammatory subsets and disease endotypes [4]. Atopy is a major risk factor for asthma, with about 30% estimated prevalence in general population from European countries, including Romania [5]. The pattern of atopic asthma is mostly based on family history of atopy, association with allergic rhinitis, high serum total immunoglobulin E (IgE), and positive skin or serum tests to the main aeroallergens.

The need for a personalized approach to asthma management is now largely known and accepted, being reinforced by actual guidelines, global initiatives, and recent studies [6-7].

## Case presentation

A 52-year-old female patient, diagnosed with adult-onset asthma in 2018, was first referred to the Allergy Department of our hospital in January 2019 for uncontrolled respiratory symptoms, consisting of dyspnea, wheezing, and worsening cough. Despite continuous inhaled combined therapy with budesonide/formoterol 320/9 ug twice daily and good inhalation technique, she had frequent asthma symptoms, requiring short courses of oral corticosteroids (CS). The physical examination on admission revealed widespread ronchi and wheezes on pulmonary auscultation, SpO2 was 92% on room air, blood pressure was 150/80 mmHg, and pulse was 88 bpm. Her body mass index (BMI) was 31.74 kg/m2. Laboratory findings showed mild systemic inflammation (slightly increased C-reactive protein) and high levels of total serum IgE (528 IU/mL, Normal <100). Allergist evaluation revealed positive skin tests and moderately increased specific IgE for dust mites and grass pollen. Pulmonary function tests confirmed moderate obstruction with a forced expiratory volume in one second (FEV1) value of 46% from predicted value, significantly reversible with bronchodilators (20% FEV1 increased). Paranasal and frontal sinuses X-ray showed opacification of the maxillary sinuses suggesting chronic sinusitis and chest X-ray was within normal limits. 

We administered short course oral CS with methylprednisolone 32 mg progressively tapered during 10 days, continued inhaled combined therapy with budesonide/formoterol 320/9 ug twice daily, and we also started oral leukotriene receptor antagonist (montelukast) and theophylline according to GINA step 4. Intranasal corticosteroid (mometasone furoat) was added for the management of chronic sinusitis.

Clinical outcome was initially good, but until the end of 2019 she had two moderate and severe exacerbations despite good adherence to therapy, requiring short courses of oral CS (methylprednisolone 32 mg progressively tapered during 10 days). We decided to start omalizumab as add-on therapy in order to reduce the rate of exacerbation and the use of oral CS. Asthma control test (ACT) questionnaire also indicates that her symptoms were not well controlled over the last month, with a score of 16. Given these clinical and laboratory findings, omalizumab dose was calculated at 525 mg subcutaneous every two weeks, according to omalizumab dose chart.

**Figure 1:**
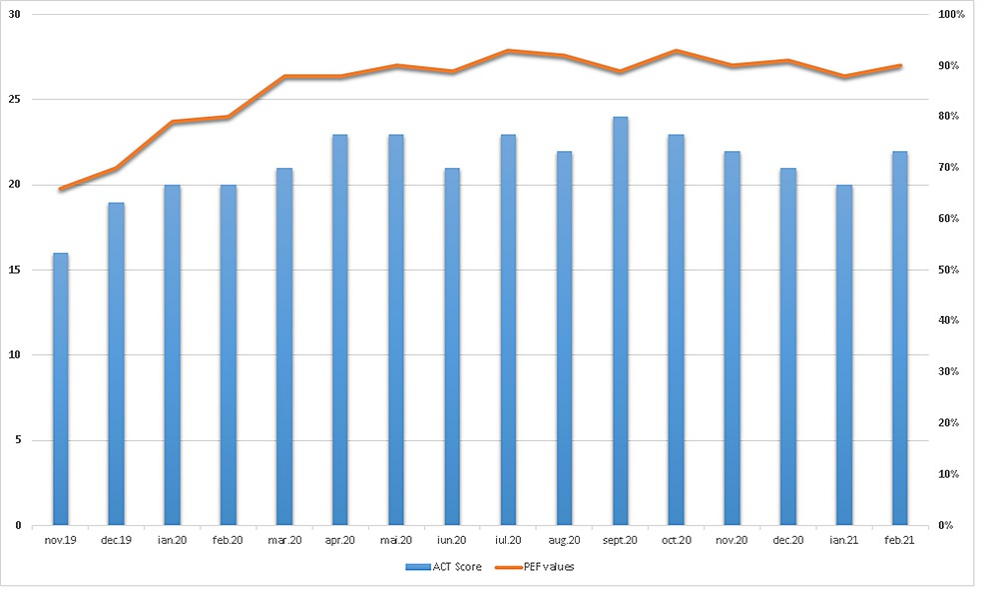
ACT score and PEF values since start of omalizumab. ACT, asthma control test; PEF, peak expiratory flow

Omalizumab was well tolerated, with significant clinical improvement, no asthma attacks, and no need for oral CS for the next six months. We continued supervised administration of omalizumab, based on hospital presentation twice a month. Since the start of COVID-19 pandemic in March 2020, we advised the patient to self-administer her subcutaneous injection of omalizumab, in order to avoid possible contact with COVID-19 patients during hospital visits. We trained the patient to record her morning peak expiratory flow (PEF) values, having a predicted 419 L/min, and we also evaluated the asthma control symptoms on phone, using ACT questionnaire, monthly. The ACT scores were constantly over 20 during the pandemic, indicating good asthma control and the PEF values were in the green zone, with no need for hospital visits. She maintained inhaled combined therapy with budesonide/formoterol 320/9 ug twice daily, and we were able to stop montelukast and theophylline after the initiation of omalizumab, six and eight months, respectively (Figure 1).

## Discussion

Diagnosis and management of asthmatic patients during COVID-19 ongoing pandemic is challenging, mainly due to the risk of contracting COVID-19 disease and limited access to hospital care and pulmonary function tests. According to recent guidelines, there is no supplementary risk of viral infection or severe clinical forms of COVID-19 disease in asthmatic patients as long as their asthma is controlled with optimal therapy [8]. The European Academy of Allergy and Clinical Immunology (EAACI) position paper has developed recommendations for the optimal management of allergy clinics during the current COVID-19 pandemic [9].

The National Institute for Health and Care Excellence (NICE) COVID-19 rapid guideline on severe asthma, recommends that patients on biological treatment should continue their treatment because there is no evidence that biological therapies for asthma suppress immunity, therefore, they do not have an increased risk of contracting COVID-19. Another recommendation of the guideline is that if the patient attends hospital for biological treatment, they should be trained to self-administer [10].

Omalizumab is the first anti-human IgE monoclonal antibody and for a long period of time was the only available monoclonal antibody for add-on therapy of severe allergic asthma [11]. It was first approved by FDA in 2003 for the treatment of moderate to severe asthma in adults and adolescents with confirmed allergy to perennial aeroallergen and whose symptoms are uncontrolled with ICS [12-13]. Omalizumab binds to free human IgE, forming small-size immune complexes that prevent interaction with the high-affinity IgE receptor (FcεR1) on mast cells and basophils. Therefore, omalizumab effectively blunts the immune response in atopic patients with asthma, significantly improving the control of asthma symptoms and the lung function. This positive effect makes it possible to decrease both referrals to the emergency room and hospitalization for asthma exacerbation [14]. 

Randomized controlled studies and real-life studies showed that omalizumab reduces the rate of asthma exacerbations, improves pulmonary symptoms and quality of life and more importantly, is able to reduce the need of oral CS [15]. Besides clinical benefits, omalizumab is characterized by a good safety profile [16].

The good selection of biologic therapy in severe asthma cases and the availability of this expensive medication are key points for achieving control of asthma, improving quality of life, and preventing long time pulmonary deterioration [17]. Early diagnosis and concomitant treatment of allergic rhinitis and rhino-sinusitis are also important. 

The role of allergist is important for good asthma management, mainly from the perspective of complete evaluation of atopy, diagnosis of respiratory allergy, concomitant therapy of pulmonary and upper airways disease, and monitoring the possible side effects of medication. The availability of the allergology specialty is different in the world and there are also some difficulties in managing allergic diseases from the perspective of ICD-10 [18]. The choice for omalizumab in our case was based on asthma phenotype, local availability, and reimbursement status of biologic therapy. 

## Conclusions

The reported case illustrates the real-life benefit of omalizumab as an add-on treatment in a patient with allergic asthma not controlled with maximal inhaled therapy, requiring frequent courses of oral CS. Moreover, with this medication we were able to maintain the control of asthma symptoms during the difficult period of COVID-19 pandemic, when access to hospital assistance was very much restricted that can be considered an even greater benefit of this biologic therapy.

## References

[1] Peters MC, Wenzel SE (2020). Intersection of biology and therapeutics: type 2 targeted therapeutics for adult asthma. Lancet.

[2] Moore WC, Bleecker ER, Curran-Everett D (2007). Characterization of the severe asthma phenotype by the National Heart, Lung, and Blood Institute's Severe Asthma Research Program. J Allergy Clin Immunol.

[3] Accordini S, Corsico AG, Braggion M (2013). The cost of persistent asthma in Europe: an international population-based study in adults. Int Arch Allergy Immunol.

[4] Leru PM (2021). Biomarkers in asthma - interpretation and utility in current asthma management. Curr Respir Med Rev.

[5] Leru PM, Kay D, Kelly J, Tuong T, Schaeffer T, Addison B, Neamtu R (2021). Atopy and lifestyle survey of allergic patients from urban environment in Romania: preliminary data from an interactive qualifying project. Cureus.

[6] (2019). Global Initiative for Asthma (GINA). Difficult-to-treat and severe asthma in adolescent and adult patients, diagnosis and management guideline. guideline.

[7] Palomares O, Untersmayr E, Gutermuth J (2020). Biologicals in allergic diseases and asthma: toward personalized medicine and precision health: highlights of the 3rd EAACI Master Class on Biologicals, San Lorenzo de El Escorial, Madrid, 2019. Allergy.

[8] (2020). British Thoracic Society. Advice for healthcare professionals treating people with asthma (adults) in relation to COVID-19. November.

[9] Pfaar O, Klimek L, Jutel M (2021). COVID-19 pandemic: practical considerations on the organization of an allergy clinic - an EAACI/ARIA position paper. Allergy.

[10] (2020). National Institute for Health and Care Excellene (NICE). COVID-19 rapid guideline: severe asthma. NICE guideline. http://www.nice.org.uk/guidance/ng166.

[11] Pelaia C, Calabrese C, Terracciano R, de Blasio F, Vatrella A, Pelaia G (2018). Omalizumab, the first available antibody for biological treatment of severe asthma: more than a decade of real-life effectiveness. Ther Adv Respir Dis.

[12] McGregor MC, Krings JG, Nair P, Castro M (2019). Role of biologics in asthma. Am J Respir Crit Care Med.

[13] Humbert M, Busse W, Hanania NA, Lowe PJ, Canvin J, Erpenbeck VJ, Holgate S (2014). Omalizumab in asthma: an update on recent developments. J Allergy Clin Immunol Pract.

[14] Snelder SM, Weersink EJ, Braunstahl GJ (2017). 4-Month omalizumab efficacy outcomes for severe allergic asthma: the Dutch National Omalizumab in Asthma Registry. Allergy Asthma Clin Immunol.

[15] Lin CH, Cheng SL (2016). A review of omalizumab for the management of severe asthma. Drug Des Devel Ther.

[16] Hanania NA, Alpan O, Hamilos DL (2011). Omalizumab in severe allergic asthma inadequately controlled with standard therapy: a randomized trial. Ann Intern Med.

[17] Agache I, Akdis CA, Akdis M (2021). EAACI biologicals guidelines - recommendations for severe asthma. Allergy.

[18] Leru PM, Deleanu DM (2015). Romanian allergology in the actual European context. Rom J Intern Med.

